# Modulation of alpha power at encoding and retrieval tracks the precision of visual short-term memory

**DOI:** 10.1152/jn.00051.2014

**Published:** 2014-09-10

**Authors:** E. Poliakov, M. G. Stokes, M. W. Woolrich, D. Mantini, D. E. Astle

**Affiliations:** ^1^Cognition and Brain Sciences Unit, Medical Research Council, Cambridge, United Kingdom;; ^2^Oxford Centre for Human Brain Activity, University of Oxford, Oxford, United Kingdom; and; ^3^Department of Experimental Psychology, University of Oxford, Oxford, United Kingdom

**Keywords:** EEG, VSTM, working memory

## Abstract

Our ability to hold information in mind is strictly limited. We sought to understand the relationship between oscillatory brain activity and the allocation of resources within visual short-term memory (VSTM). Participants attempted to remember target arrows embedded among distracters and used a continuous method of responding to report their memory for a cued target item. Trial-to-trial variability in the absolute circular accuracy with which participants could report the target was predicted by event-related alpha synchronization during initial processing of the memoranda and by alpha desynchronization during the retrieval of those items from VSTM. Using a model-based approach, we were also able to explore further which parameters of VSTM-guided behavior were most influenced by alpha band changes. Alpha synchronization during item processing enhanced the precision with which an item could be retained without affecting the likelihood of an item being represented per se (as indexed by the guessing rate). Importantly, our data outline a neural mechanism that mirrors the precision with which items are retained; the greater the alpha power enhancement during encoding, the greater the precision with which that item can be retained.

the ability to hold in mind small amounts of visual information for brief periods of time is critical for a wide variety of everyday cognitive tasks. Importantly, this ability is limited, resulting in an ongoing debate as to the nature of resource allocation within visual short-term memory (VSTM; Bays and Husain 2008; Wilken and Ma 2004; Zhang and Luck 2008). Some researchers have argued that resources can be allocated such that the quality of item representation is reduced as the number of to-be-represented items increases, indicative of a flexible capacity limit. By contrast, others have argued that a limited number of fixed resolution item representations can be maintained. Evidence from a particular paradigm has been informative in this debate: participants are presented with memoranda that can be varied in a continuous way, such as hue (Zhang and Luck 2008) or line orientation (Bays and Husain 2008), and by providing participants with means of freely recalling a cued item (for instance, using a color wheel or dial, respectively). This method of free recall enables the researcher to assay the underlying content of VSTM using a model-based approach (e.g., Anderson and Awh 2012; Anderson et al. 2013; Bays et al. 2009, 2011). In particular, it is possible to estimate the proportion of trials on which participants correctly retain a representation of a target item (and the precision with which they do so), those on which they incorrectly retain a nontarget representation, and the proportion of trials on which participants simply guess.

However, it remains unclear when these resources are allocated within VSTM (whether they are discrete or flexible). These resource limitations could be imposed when we encode items and maintain them within VSTM or when we attempt to retrieve them for report. We address this using a paradigm in which we combine the sequential presentation of items and a retrieval cue, alongside EEG recording, to isolate the different phases of a trial.

In a parallel literature to that exploring resource limitations within VSTM, a number of research groups have used magnetoencephalography (MEG) or EEG to study the role of neural oscillations in VSTM processes. Modulations in the alpha band are consistently associated with changes in VSTM performance such as load effects or retrieval success (see Klimesch 1999 for review). Recently, Myers et al. (2014) demonstrated that spontaneous fluctuations in alpha band activity up to and around the onset of potential memoranda modulated the encoding of that item. Relative alpha desynchronization improved the encoding of subsequent items, whereas relative synchronization decreased the interference of distracting items. Furthermore, these rhythms appear to be causally related to the subsequent retrieval success; enhancing the alpha rhythm with transcranial magnetic stimulation (TMS) can result in enhanced suppression of irrelevant information contralateral to the stimulated hemisphere (Sauseng et al. 2009). Indeed, there is an emerging consensus that modulations in the alpha rhythm provide a mechanism for functional inhibition and that top-down changes in alpha amplitude and phase can facilitate goal-directed behavior by gating sensory information. Romei et al. (2010) demonstrated that alpha band activity is inversely related to perceptual sensitivity with peristimulus alpha suppression resulting in improved perception. One possible explanation for the relationship between alpha band modulation and VSTM performance is because the former is a mechanism of attentional control, variability in which drives differences in VSTM task performance.

The study of neural oscillations has only just begun to be combined with a paradigm that allows for a continuous method of responding. It is as yet unclear which neural mechanisms influence the accuracy with which participants can maintain items in VSTM and when this influence occurs. For example, variability in the neural mechanisms applied during encoding could predict the variability with which items are retrieved subsequently. Alternatively, the variability in VSTM accuracy could be driven by neural mechanisms applied as participants attempt to retrieve the items from memory. Of course, these possibilities are not mutually exclusive. Here, we use a cueing paradigm to disentangle item encoding and retrieval from each other and from response-related effects, and this is combined with an analysis approach that enables us to establish the relationship between neural oscillations and trial-to-trial variability in VSTM accuracy (absolute circular error).

As well as testing which period of processing is critical for determining the accuracy with which items can be retrieved, we also used a modeling approach to explore how this neural mechanism influences performance. One possibility is that these mechanisms influence the likelihood of an item successfully being retained in, and retrieved from, VSTM, i.e., these mechanisms may effectively increase the likelihood that the probed item is represented in VSTM. Alternatively, these mechanisms may have little influence on the likelihood of an item being retained per se but instead boost the precision with which successfully maintained items can be remembered. In a subsequent analysis, we use a model-based approach to distinguish between these two possibilities. In summary, we were able to identify when particular neural oscillations would predict the accuracy of participants' retrieval, and then we used a modeling approach to disentangle the different behavioral effects of this mechanism.

## METHODS

### 

#### Subjects.

Fourteen subjects each completed an EEG session (4 men and 10 women; aged 21–29 yr). All reported normal color vision and normal or corrected-to-normal visual acuity. Subjects provided informed, written consent and received monetary compensation (£20). This experiment was approved by the Psychology Research Ethics Committee at the University of Cambridge.

#### Behavioral task.

On each trial, participants were presented with a sequence of randomly oriented arrows to be remembered. Each arrow was a different color and was surrounded by a distracter ring, which was itself colored and had orientated lines running through it. Following this, a colored disc was presented at fixation corresponding to the color of the target arrow that subjects ought to retrieve from memory. This colored disc is essentially a feature-based retrospective cue. Following this, a white arrow was presented at fixation, which subjects could rotate until its orientation matched their memory of the cued target arrow. After submitting the response, subjects were given feedback on how accurate they were.

#### Task design.

**Fig. 1.**
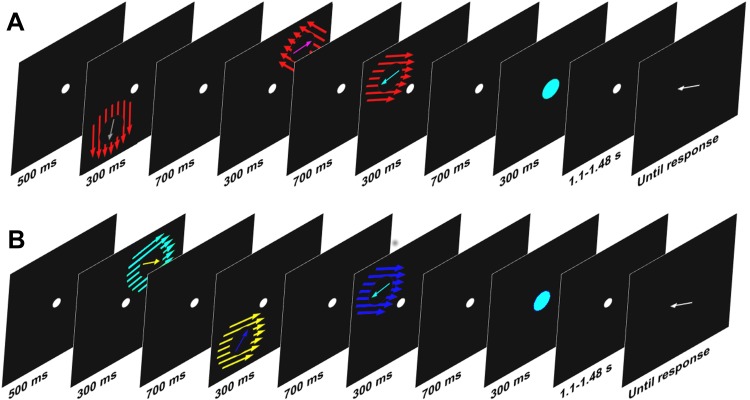
A trial schematic showing the onset of the 3 target items and their accompanying surrounds followed by the retrieval cue and the white arrow for subjects to indicate their response. *A* shows a trial wherein the surrounding distracter ring is of a different color from all of the targets on that trial. *B* shows a trial wherein each of the surrounding distracters shares its color with 1 of the other targets from that trial.

The experiment consisted of 7 blocks of 30 trials, and subjects were given 30 practice trails before the experiment. In an additional manipulation, we varied the color of the distracter rings. For half of the trials, each of the 3 distracters on that trial was of the same color, and this was different from all target arrow colors for that trial (Fig. 1*A*). For the other half of trials, each distracter shared the color of 1 of the target arrows from that trial. To be clear, each target was never presented alongside the distracter of the same color (Fig. 1*B*). These 2 types of trial were interleaved. We added in these trials with distracters that matched the target colors to test 1 supplementary prediction, that subjects would be more likely to report distracters erroneously if they shared a feature (namely color) with the target. Although to foreshadow the results, this manipulation had no impact on the likelihood of subjects reporting the distracter.

Every arrow, distractor ring, and final white arrow was assigned a random orientation from the set of multiples of 4°. All arrows and rings had one of seven highly discriminable colors. Six colors where chosen from the eight vertices of the RGB cube (excluding black for background and white for the reporting arrow), and the seventh was taken from the middle of the cube (RGB 128, 128, 128) as follows: yellow, green, red, pink, light blue, dark blue, and gray.

In all of our trials, the set size was three items. Every trial (Fig. 1*A*) began with a white fixation dot presented for 500 ms. Then, three arrows inside distractor rings followed for 300 ms each, separated by 700-ms delays (breaks of blank screen with fixation dot). The three arrows were positioned at three (randomly chosen for each trial) vertices of the invisible square (diagonal 10°). The length of the arrow was 0.7°, and the diameter of the distractor ring 1.7°. Then, the retrospective cue was presented in the middle of the screen for 300 ms (diameter 0.9°). A blank screen with fixation dot followed for a randomly chosen time between 1,100 and 1,480 ms before a white arrow appeared in the middle of the screen cueing the subject to report the target orientation.

Subjects used keyboard buttons to report the orientations of the target arrow by rotating the white arrow in a clockwise or anticlockwise direction in small steps (4°) and big steps (12°). Subjects were encouraged to use both of these step sizes to fine-tune their responses in a timely manner. After reporting the target arrow, subjects were given the accuracy feedback for 1,000 ms (degrees of the angle between target arrow and their response arrow). Participants performed the computerized task in a silent, dimly lit room viewing the cathode-ray tube (CRT) monitor at a distance of 130 cm. Accuracy was stressed, and the responses were not timed.

#### Behavioral data analysis.

We only included trials in the behavioral analysis that were also included in the EEG analysis. First, this meant that we could easily relate the two sets of analysis. Second, those artifactual trials removed from the EEG analysis, due to large muscle artifact or excessive noise from subjects having moved, if included, may well have resulted in us overestimating the true guessing rate by including some trials wherein the item was missed for noncognitive reasons. On average, 16.8% (± 7.06 SD) trials were removed because of poor EEG quality.

We analyzed subjects' responses with respect to the line orientation. Initially, this was done by calculating the overall circular error in response angle. From this, we calculated the overall precision (the inverse of the SD of the circular error) with which subjects responded and then used a modeling approach to explore subjects' responses more closely. The distribution of responses (using a mixture model from Bays et al. 2009) was divided into two parts: a von Mises (a circular Gaussian distribution) around the target arrow (a correct item being retrieved) and a uniform distribution (random guess). This modeling provides values for each subject as to the proportion of trials making up these two distributions. An alternative suitable model would have been the variable-precision model (Fougnie et al. 2012; van den Berg et al. 2012) as recent studies have shown that in some cases this can provide a better account of data of this sort. This is also a mixture model but allows for precision to vary across trials with the precision estimate being expressed as two parameters, the mean SD of the circular error (mnSD) and the SD of that value across trials (stdSD). Because the trial numbers are relatively low in this study, we could not distinguish which of the two models provided the best fit for the data: using the MemToolbox (Suchow et al. 2013) to simulate data with the variable-precision model and then perform a formal model comparison between this model and the standard mixture model, we found that the latter was the preferred model (according to the Akaike information criterion) on 16 out of 20 iterations. In other words, even when the data are created using the variable-precision model, because the trial numbers are relatively low, the simpler mixture model wins more often than not. Because we could not distinguish reliably between these two models in this experiment, we used the standard mixture model for subsequent analyses but also checked any significant effects with the variable-precision model to test whether the two models converge on the same result.

We also used a three-factor version of the standard mixture model to test one supplementary hypothesis, that subjects would be more likely to report distracter items erroneously when they share a feature (namely color) with the target. To do this, we used the trials wherein each potential target shared a feature with one distracter ring (Fig. 1*B*). We modeled the data first using the angle of the distracter ring that matched that of the target and then using the angle of the other distracter ring (that did not match the color of the target). These values were added to a version of the model that incorporated the angles of additional nontarget items. In no cases did we use the distracter ring that was presented alongside the target. We then compared the various parameters produced by these two models focusing specifically on the estimated likelihood of subjects reporting the distracter.

The standard mixture model can be described by the following equation (Bays et al. 2009, 2011):

P(θ^)=αϕk(θ^−θ)+β1m∑imϕk(θ^−φi)−γ12π,

with θ corresponding to the correct orientation of the target item and θ̂ being the orientation reported by the subject. $\phi_{k}$ Corresponds to the von Mises distribution (a Gaussian distribution equivalent for a circular response space) with a mean of 0 and concentration *k*. α Is the probability of reporting the target item; β is the probability of reporting a nontarget item with *m* corresponding to the number of potential nontarget items. The probability of responding randomly can be calculated using γ = 1 − α − β. The main model that we used only comprised the α and γ terms (i.e., just the target and uniform distributions), but any models including nontarget angles include the β term. Further details on the implementation of this mixture model can be found at http://www.paulbays.com/code/JV10/.

The mixture model was applied for each subject, and four estimates were calculated: kappa, the concentration coefficient of the von Mises distribution; T_p_, the proportion of responses to the target; U_p_, the proportion of random guesses; and, where appropriate, N_p_, the proportion of responses to the nontargets. The concentration coefficient of the von Mises distribution is a measure of the height of the distribution and was therefore used as a measure of the precision with which target arrows were reported.

#### EEG preprocessing.

EEG activity was recorded continuously using a BrainVision amplifier and actiCAP electrodes mounted on an elastic cap from 32 sites according to the 10-20 system. The montage included 4 midline scalp sites (Fz, Cz, Pz, and Oz) and 13 scalp sites over each hemisphere (FP1/FP2, F7/F8, F3/F4, FC1/FC2, FC5/FC6, T7/T8, C3/C4, CP1/CP2, CP5/CP6, TP9/TP10, P3/P4, P7/P8, PO9/PO10, and O1/O2). AFz served as the ground. Blinks and eye movements were monitored with electrodes placed horizontally and vertically around the eyes. Electrode impedances were kept below 20 kΩ. We used a 250-Hz analog-to-digital sampling rate and recorded all frequencies between 0.1 and 124 Hz. The EEG was referenced online to the FCz electrode and then rereferenced offline to the algebraic average of the left and the right mastoids. Bipolar electrooculogram (EOG) signals were derived by computing the difference between recordings horizontal to each eye (HEOG) and between recordings vertical (VEOG) to the left eye. Eye movements and blinks were removed using an independent component analysis (ICA): we applied a 1-Hz high-pass filter and submitted the continuous EEG to a temporal ICA (using EEGLAB; Delorme and Makeig 2004); we correlated the time course of each IC with our bipolar EOG channels to identify the ICs that corresponded to blinks and eye movements by manual identification; these were then regressed from the data. We also used the FieldTrip visual artifact rejection tool to remove any trials that contained large artifacts not corrected for by the temporal ICA. Other than those described above, no further filters were applied. We extracted amplitude estimates from the EEG data using a continuous wavelet transform (Kronland-Martinet et al. 1987). This used six full cycles to establish the phase angles and power estimates for frequencies in the alpha (8- to 10-Hz) range. We performed our subsequent analyses on these frequencies because they have been regularly associated with VSTM processes (Jensen et al. 2002; Shimi and Astle 2013).

#### Event-related alpha modulation analysis.

We wanted to determine the neural signatures that reflect memory accuracy during encoding and/or retrieval. To do this, rather than averaging across trials, we used the absolute circular error term (subjects' response angle on that trial minus the correct angle) for each individual trial as a continuous regressor. To aid interpretation, we inverted this regressor so that higher values corresponded to more accurate recall (i.e., better VSTM) rather than a larger error. This regressor was included in a general linear model (GLM), otherwise known as a multiple regression analysis. This GLM was applied to the data over trials separately for each time sample, electrode, and participant. The result was a data set in which we could establish the linear effects of the regressor (β-coefficients), over and above a constant term, including their topographical distribution and time course, within each participant. These were then fed into a group-level mixed-effects analysis applied to the β-coefficients resulting from the GLM on each subject, separately for each time sample and electrode, allowing us to identify significant effects of the regressor at the population level. To do this, we used slightly longer epochs than needed (−400 to 1,000 ms) so as to avoid edge effects in our time-frequency decomposition. Our GLM contained a regressor that corresponded to the effect of trialwise accuracy on target-locked epochs, a separate regressor corresponding to cue-locked epochs, and two further regressors that corresponded to the mean of each epoch type. We then explored the effect of the first two regressors by passing the within-subject effects of each into a group-level analysis that estimated their effect at the population level.

Once the group-level analyses were completed, we identified clusters of consecutive samples (contiguous in time and/or across neighboring electrodes). First, we identified the size of each cluster with all of the *t* values in that cluster exceeding a fixed threshold (*t* > 3.012). We chose this level because it corresponds to an uncorrected *P* value of 0.01 with a sample of 14 subjects. Next, we used a sign-flipping permutation procedure to produce a null distribution using 5,000 permutations. With each random permutation, we identified the size of any clusters where *t* > 3.012 to determine the distribution of cluster sizes expected by chance under the null hypothesis. We were then able to compare the size of our observed clusters to this null distribution thereby deriving a probability value (*P*). In essence, this procedure estimates the likelihood of us observing a cluster of contiguous *t* values (across adjacent electrodes and time points), all of which are >3.012, in random data and expresses this likelihood as a *P* value (Nichols and Holmes 2002). This approach has a number of advantages relative to more traditional approaches to significance testing with electrophysiological data: first, it makes no a priori assumptions about when or where effects are likely to be apparent, as is sometimes the case if researchers focus on particular peaks and latencies; second, this approach accounts for multiple comparisons over space and time, which can result in reporting spurious effects if not corrected for (Kilner 2013).

A similar type of analysis could be run using simple correlations rather than a multiple regression. An advantage of using the latter is that it allows us to incorporate multiple repressors, such as those representing the trial mean in this EEG analysis, while also accounting for normally distributed error or noise.

## RESULTS

### 

#### Behavioral results.

**Fig. 2.**
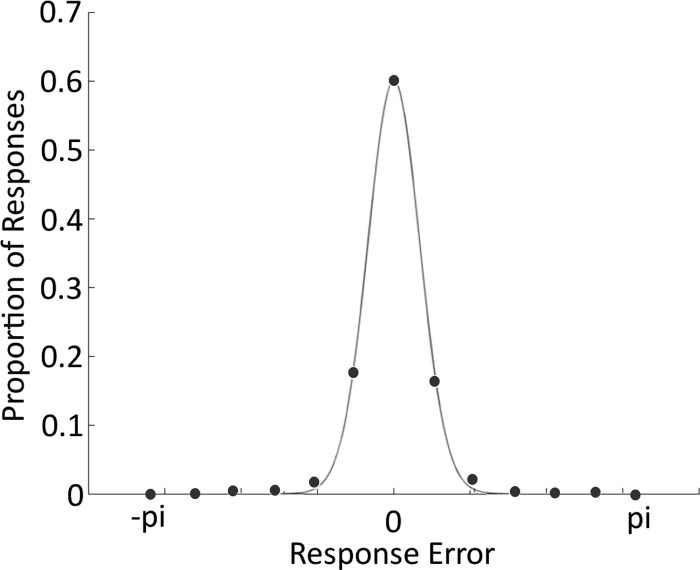
The mean distribution of errors across 13 equally spaced bins.

The trial-by-trial responses were mapped onto a circular distribution such that all fell within the range −π < *x* < π. We then calculated the difference between the target and response values on each trial. For each subject, we then produced a histogram, showing the proportion of responses within 13 equally spaced bins spanning the possible range of errors. The mean proportion of responses within each bin across all subjects can be seen in Fig. 2; subjects responded with a mean precision of 2.81 (the inverse of the SD of the circular error) with a SD of 0.82.

The mixture model that distinguished the target and uniform distributions demonstrated that 97.64% of participants' responses were to the target (with the remaining 2.36% of responses being attributed to guessing). The concentration (or kappa) of those target responses was 13.52. Note that in all cases the model was applied to single subjects and then averaged across subjects.

We also used this modeling approach to test whether distracters that share a common feature with the target are more likely to be reported than those that do not. In modeling terms, this would result in a higher N_p_ for distracters of the same color, relative to those of a different color. We tested this using the trials wherein targets and distracters could share their color and modeled separately the various parameters using the nontarget angles from the target-colored distracters and then the angles from the nontarget-colored distracters. However, none of the parameters produced by the mixture model differed significantly across these two contexts (all *P*s > 0.1). In short, when cued with a color, subjects were no more likely to recall erroneously and report the distracter of that color.

#### Event-related alpha modulation analysis.

**Fig. 3.**
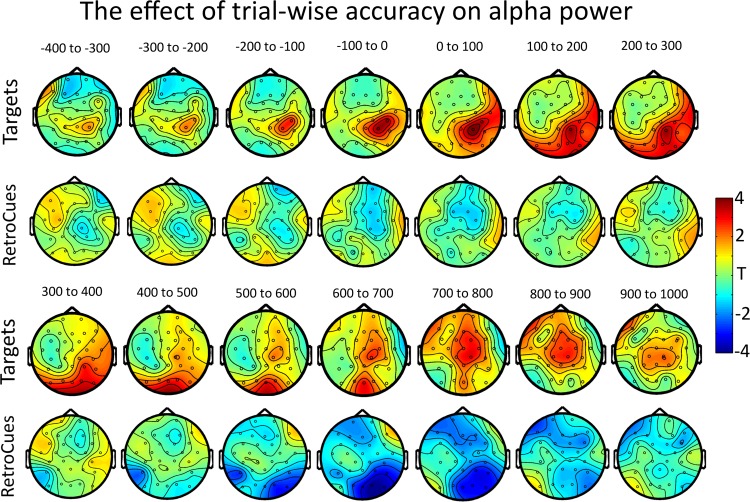
The topographical distribution of the relationship between trial-to-trial accuracy and alpha power. This is shown across 100-ms bins starting before the onset of the target (*top* rows) or retrospective cue (*bottom* rows).

**Fig. 4.**
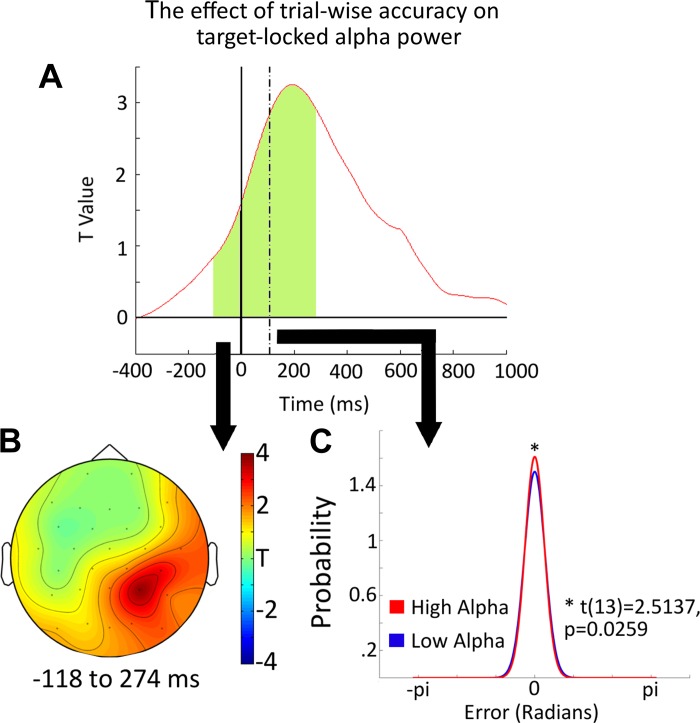
*A*: the time course of the relationship between trial-to-trial variability in accuracy and target-locked alpha band power as produced by our general linear model (GLM) analysis. The effect is shown at PO10, O2, and PO8. The green shaded area shows the time window of significance, counted as the earliest onset of the cluster at any electrode to the latest offset at any electrode within the cluster. *B* shows the topographical distribution of the effect of trialwise accuracy on alpha power, between −118 and 274 ms, relative to the onset of the target item. *C* shows the von Mises distribution of response on high and low alpha trials, depicted as a probability density function, and the asterisk indicates the significant differences in the concentration of these responses.

Around the onset of the target, activity in the alpha band, starting before the onset of the target (−112 ms) and ending briefly before its offset (274 ms), was related significantly to the accuracy with which that item would be subsequently retrieved. The greater the alpha synchronization at PO10, PO8, and O2, the better the subsequent accuracy (*P*_corrected_ = 0.0414). The distribution of this effect throughout the epoch, in 100-ms bins, can be seen in Fig. 3. The time course of the effect, alongside the topography of the significant time window (taken as the earliest value greater than *t* = 3.012 at any electrode within the cluster to the last value greater than *t* = 3.012 within the cluster), can be seen in Fig. 4, *A* and *B*, respectively.

**Fig. 5.**
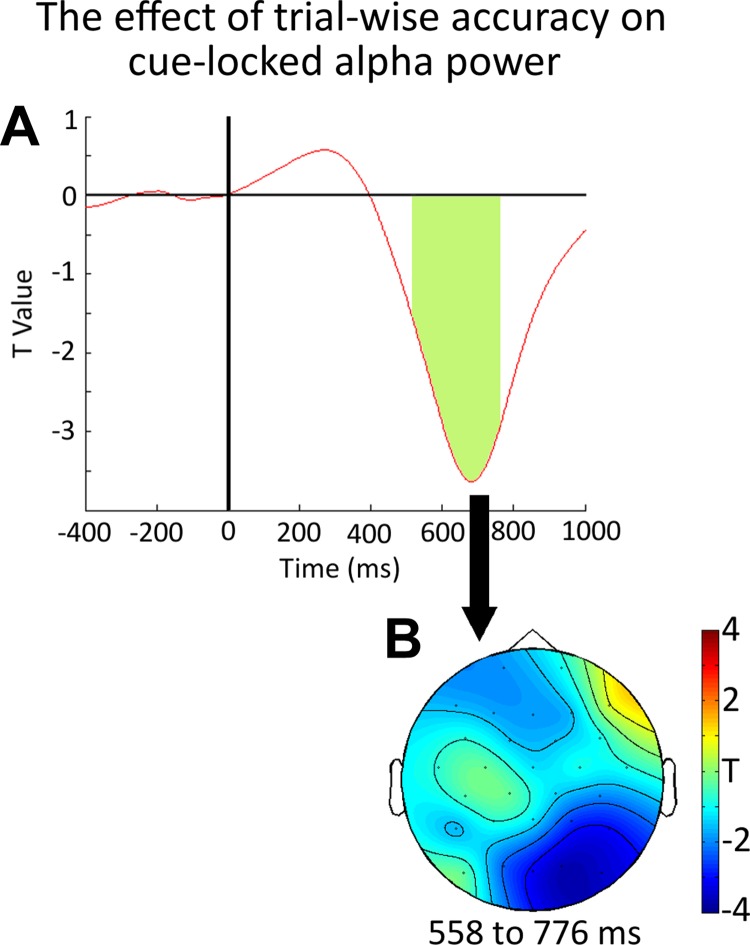
*A*: the time course of the relationship between trial-to-trial variability in accuracy and retrospective cue-locked alpha band power as produced by our GLM analysis. The effect is shown at PO10, O2, Oz, P4, and P8. The green shaded area shows the time window of significance, counted as the earliest onset of the cluster at any electrode to the latest offset at any electrode within the cluster. *B* shows the topographical distribution of the effect of trialwise accuracy on alpha power, between 558 and 776 ms, relative to the onset of the retrospective cue item.

Later, during the retrieval period, as subjects attempted to use the retrospective cue to retrieve the item from VSTM, alpha band desynchronization was significantly related to subsequent accuracy (558–776 ms; *P*_corrected_ = 0.0358). Again, the distribution of this effect, throughout the epoch, in 100-ms time bins, can be seen in Fig. 2. The time course of the effect, and the topography of the significant window, can be seen in Fig. 5, *A* and *B*, respectively.

#### Modeling the behavior from high and low alpha trials.

Given that peristimulus alpha synchronization and cue-locked alpha desynchronization predict the subsequent accuracy with which that target can be retrieved, we tested in more detail how alpha band activity modulates subsequent performance. One possibility is that the alpha modulation improves the precision of the successfully maintained targets, i.e., it is not that more items are retained per se but those that are retained are done so with an improved precision. Alternatively, the alpha modulation may increase the discrete probability that an item will be recalled (thereby reducing the guess rate). In modeling terms, the former account would result in a relationship between alpha band activity and the kappa parameter. By contrast, the latter account would result in a relationship between alpha band activity and U_p_ (the proportion of guesses).

We isolated the raw alpha amplitudes corresponding to each of our significant effects. To do this, we extracted the power values for the electrodes and time points identified by our GLM analysis. Within this window, we identified the peak time of the GLM effect for each subject separately for the target and cue-locked effects. We used these values to divide the data into high and low alpha trials using a median split. The performance on these two classes of trial was then modeled separately for each participant. For trials split according to the target-locked effect, high alpha trials had greater precision (kappa) than did lower alpha trials [16.55 vs. 14.43, respectively; *t*(13) = 2.5137, *P* = 0.0259], but there was no difference in U_p_ [2.5 vs. 2%, respectively; *t*(13) = 0.5752, *P* = 0.5750]. This precision effect can be seen in Fig. 4*C*. We also checked this comparison by fitting the variable-precision model. This is a more sophisticated version of the standard mixture model, allowing precision to vary across trials. As such, it produces an estimate of U_p_ but splits the estimate of precision into two parameters. The first parameter expresses the average precision across trials as the mnSD. The second parameter expresses the stdSD. Using this model instead of the standard mixture model produces the same result. Responses are significantly less variable (i.e., more precise) when target-locked alpha power is high relative to when it is low [mean mnSD = 13.98 vs. 16.55°, respectively; *t*(13) = 3.302, *P* = 0.005]. The other two parameters from the model were unaffected [mean mnSD = 6.19 vs. 5.07°, respectively; *t*(13) = 0.767, *P* = 0.458; mean Tp = 1.2 vs. 1.6%, respectively; *t*(13) = 0.485, *P* = 0.636]. We also checked whether the same result could be achieve with no model at all, simply comparing the precision values (the inverse of the SD) without using a model to separate the guesses and the target responses. Although the difference is in the direction we would expect, with responses being more precise on high relative to low alpha trials, this does not reach significance [2.99 vs. 2.68; *t*(13) = 1.896, *P* = 0.08]. This demonstrates that the use of the modeling (either the variable-precision model or the standard mixture model) to separate the target responses and guesses does provide greater sensitivity to detect changes in VSTM precision.

When trials were split by the cue-locked effect, we could find no reliable changes in either response precision [kappa; *t*(13) = 0.5392, *P* = 0.5995] or Tp [*t*(13) = 1.5180, *P* = 0.1530].

## DISCUSSION

Subjects were presented with a set of to-be-remembered arrows embedded among distracters. One target was subsequently cued with a retrospective feature cue and had to be recalled using a free-recall paradigm. Our data demonstrate that alpha band activity was modulated depending on the accuracy (absolute circular error) with which target items could be subsequently reported. Peristimulus alpha activity was enhanced during encoding, and we also observed alpha suppression during the retrospective cue period, both of which were related to subsequent VSTM accuracy. In short, trial-to-trial performance variability in our paradigm was significantly related to fluctuations on alpha band activity at both encoding and retrieval. There have been a number of demonstrations that alpha power over visual areas can provide an index of cortical excitability, which corresponds to enhanced sensory gain. This is most clearly seen when items are at the limits of what is perceptible (Thut et al. 2006). Moreover, alpha band activity has also been shown to modulate memory maintenance: using lateralized TMS to enhance the alpha rhythm during VSTM maintenance can facilitate the inhibition of contralateral to-be-ignored items and thereby improve the retrieval of ipsilateral to-be-remembered items (Sauseng et al. 2009).

Recent findings in the nonhuman primate literature demonstrate that levels of alpha activity are inversely related to neuronal firing rate. When alpha levels are high, this has a suppressive effect on spike activity, thereby reducing cortical excitability (Haegens et al. 2011). For this reason, modulations of alpha power provide a good candidate by which attentional biases might operate at a cortical level, with pools of neurons being suppressed or excited according to the relevance of their preferred stimuli to the task at hand.

More recently, Myers et al. (2014) have shown that spontaneous fluctuations in alpha power before the presentation of a VSTM item predicts the likelihood of successful encoding and/or retrieval. Similarly, when subjects anticipate a distracter, alpha amplitudes are enhanced leading up to and around the onset of the item. This has also been shown with phase alongside power modulations, with a number of researchers suggesting that modulations of the alpha rhythm might provide a mechanism by which subjects can protect VSTM maintenance against the interference of distracting information (Bonnefond and Jensen 2012, 2013; Payne et al. 2013). Particularly relevant here, alpha band activity can also be enhanced when to-be-remembered targets are embedded among to-be-ignored distracters depending on how perceptually similar the distracters are to the relevant target (Shimi and Astle 2013). Recent evidence has taken this approach a step further by suggesting that activity within the alpha band may index the actual content of VSTM (in terms of line orientation) and not just its quality (Anderson et al. 2014).

Our findings extend these previous demonstrations of the relationship between alpha power and VSTM maintenance. Using a novel regression-based approach, we show that graded changes in the posterior alpha power, around the onset of the item and during its attempted retrieval, predict the absolute circular accuracy with which that item can be reported at the end of the trial. The former effect is related to the precision (kappa) with which the item is stored rather than the discrete probability of the item being stored per se (T_p_). Importantly, this conclusion is also supported by a model that allows for precision to vary across trials. We suggest that this is because this alpha band activity gates the perceptual formation of representations and thus limits the subsequent quality with which they can be maintained in VSTM. When the targets are embedded among salient distracters, alpha band activity can be enhanced to facilitate the selective processing of the memoranda (see also Shimi and Astle 2013). When subjects use the retrospective cue to prepare themselves for reporting the target, alpha power suppression may act to enhance the fragile representation of that particular item within VSTM such that it survives the subsequent delay and onset of other stimuli. What we cannot currently distinguish is whether these alpha band changes are common to all targets within a trial or specific to just the one probed. It could be that the trial-by-trial target-locked effects that we observe correspond to a more general state of readiness that spans a whole trial, or they could correspond to a more stochastic effect that can change within a trial. Likewise, one possibility is that the alpha band modulation is retinotopically organized, indicating that it corresponds to a relatively low level of item representation, vs. being spatially nonspecific. Future studies could explore systematically the relationship between target location and the topographical distribution of the alpha modulation.

In this study, we did not vary the number of memoranda, but it will be important to adopt a similar analytic approach to that used here to explore the neural mechanisms that mirror the trade-off between VSTM load and precision. One possibility is that alpha band activity represents a mechanism by which subjects vary the resolution with which they maintain items. However, this is not to imply that this trade-off is under volitional control (Murray et al. 2012). Varying the VSTM load would also be important for checking that the relationship between alpha power and the modeling parameters is constant across the different levels of load. It could be that when load is higher, and the guessing rate increased, we might see a relationship between Tp and alpha power, even though we did not observe one here. Furthermore, additional mechanisms, potentially at frequency bands not explored here, might index the discrete number of items maintained (Freunberger et al. 2011).

Finally, an important difference in this study, relative to previous uses of cognitive modeling to explore responses on similar free-recall tasks, is that we used the EEG data to exclude trials from the behavioral analysis. We think that this is important because including trials in which subjects moved or did not fixate the target items will result in an enhanced guessing rate (i.e., uniform distribution). This could be erroneously attributed to a characteristic limit of VSTM, but it instead stems from poor data quality. These trials also tend to result in large EEG artifacts, and so their removal might contribute to why our guessing rate is lower than other previously reported studies (e.g., Bays et al. 2009). It is also important to note that we gave participants a retrospective cue, providing them with warning of the item that they were subsequently to report, before the onset of the probe stimulus. This, too, could have contributed to the improved performance that we noted (e.g., Astle et al. 2012a,b; Murray et al. 2013).

In conclusion, we demonstrate that alpha band modulation both during item encoding and at retrieval is significantly related to the accuracy with which VSTM items are reported. The use of mixture modeling indicates that at encoding this alpha modulation acts to make the item representation more precise rather than by altering the discrete probability that it is maintained.

## GRANTS

D. E. Astle was supported by a British Academy Postdoctoral Fellowship and by the Medical Research Council (United Kingdom) intramural program (MC-A060-5PQ40), which provided funding to pay the Open Access publication charges for this article. M. W. Woolrich was funded by the Wellcome Trust and the Medical Research Council-Engineering and Physical Sciences Research Council United Kingdom MEG Partnership Award. M. W. Woolrich and M. G. Stokes were supported by the National Institute for Health Research (NIHR) Oxford Biomedical Research Centre based at Oxford University Hospitals National Health Service (NHS) Trust [the views expressed are those of the author(s) and not necessarily those of the NHS, the NIHR, or the Department of Health]. D. Mantini is supported by a Sir Henry Dale Fellowship jointly funded by the Wellcome Trust and the Royal Society (Grant 101253/Z/13/Z).

## DISCLOSURES

No conflicts of interest, financial or otherwise, are declared by the author(s).

## AUTHOR CONTRIBUTIONS

E.P. and D.E.A. conception and design of research; E.P. and D.E.A. performed experiments; E.P., M.W.W., D.M., and D.E.A. analyzed data; E.P., M.G.S., and D.E.A. interpreted results of experiments; E.P. and D.E.A. prepared figures; E.P. and D.E.A. drafted manuscript; E.P., M.G.S., M.W.W., D.M., and D.E.A. edited and revised manuscript; E.P., M.G.S., M.W.W., D.M., and D.E.A. approved final version of manuscript.
